# Twitter social bots: The 2019 Spanish general election data

**DOI:** 10.1016/j.dib.2020.106047

**Published:** 2020-07-21

**Authors:** Javier Pastor-Galindo, Mattia Zago, Pantaleone Nespoli, Sergio López Bernal, Alberto Huertas Celdrán, Manuel Gil Pérez, José A. Ruipérez-Valiente, Gregorio Martínez Pérez, Félix Gómez Mármol

**Affiliations:** a Department of Information Engineering and Communications, University of Murcia, Murcia, Spain; b Telecommunication Software & Systems Group, Waterford Institute of Technology, Cork Rd, Waterford, Ireland

**Keywords:** Social bots detection, Social bots classification, Machine learning, Sentiment analysis, Social network analysis

## Abstract

The term social bots refer to software-controlled accounts that actively participate in the social platforms to influence public opinion toward desired directions. To this extent, this data descriptor presents a Twitter dataset collected from October 4th to November 11th, 2019, within the context of the Spanish general election. Starting from 46 hashtags, the collection contains almost eight hundred thousand users involved in political discussions, with a total of 5.8 million tweets. The proposed data descriptor is related to the research article available at [1]. Its main objectives are: i) to enable worldwide researchers to improve the data gathering, organization, and preprocessing phases; ii) to test machine-learning-powered proposals; and, finally, iii) to improve state-of-the-art solutions on social bots detection, analysis, and classification. Note that the data are anonymized to preserve the privacy of the users. Throughout our analysis, we enriched the collected data with meaningful features in addition to the ones provided by Twitter. In particular, the tweets collection presents the tweets’ topic mentions and keywords (in the form of political bag-of-words), and the sentiment score. The users’ collection includes one field indicating the likelihood of one account being a bot. Furthermore, for those accounts classified as bots, it also includes a score that indicates the affinity to a political party and the followers/followings list.

Specifications table

**Table:** 

Subject	Computer Science
Specific subject area	Artificial Intelligence
Type of data	MongoDB BSON, JSON, CSV
How data were acquired	Social Feed Manager [2], Twitter API [3], Botometer API [4]
Data format	Anonymized raw
Parameters for data collection	The harvester collected data from 04/10/2019 to 11/11/2019. Each retrieved tweet matches at least one hashtag among the ones selected and described in Table 3. The bot score threshold to separate the human accounts from the social bots has been selected as the 95th percentile (i.e., 0.69).
Description of data collection	The observation period spans from 04/10/2019 to 11/11/2019. In this time frame, Twitter APIs have been used to retrieve tweets matching a list of 46 hashtags and keywords. A recursive search completes the missing referenced tweets. For each unique user, the framework queried Botometer for collecting the users’ bot score. For those identified as bots, we also collected their followers and friends lists.
Data source location	Department of Information and Communications Engineering, University of Murcia (Spain)
Data accessibility	Data repository: Spotting political social bots in Twitter: A dataset for the 2019 Spanish general election [5]. Data identification number: 10.17632/6cmyyxswyp Direct URL to data: http://dx.doi.org/10.17632/6cmyyxswyp Source code repository: Botbusters - Analysis of the 2019 Spanish general election [6] Source code URL: https://github.com/CyberDataLab/botbusters-spanish-general-elections
Related research article	J. Pastor-Galindo, M. Zago, P. Nespoli, S. López Bernal, A. Huertas Celdrán, M. Gil Pérez, J.A. Ruipérez-Valiente, G. Martínez Pérez, F. Gómez Mármol, 2020. Spotting political social bots in Twitter: A use case of the 2019 Spanish general election. Preprint arXiv:2004.00931 [1]

## Value of the Data

• This dataset aims to overcome one of the literature challenges on social bot detection, the scarce presence of recent data regarding bots activity, and, on the other hand, it also intends to investigate the presence, the activity, and the possible influence of social bots in the 2019 Spanish general elections.
• The principal beneficiaries of the proposed dataset are the worldwide researchers that are studying the social bots phenomenon and, particularly, its implications in the political context.
• This dataset can be highly useful for the scientific community to test and propose machine learning solutions to eventually move beyond the state-of-the-art proposals in the social bots detection ecosystem. These data can also help to understand the role of bots in modern politics.
• These data, methodologies, and code sources are distributed under an open license. To this extent, we ensure essential properties such as the replicability, comparability, and testability of each component.

## Data description

The dataset consists of two collections, specifically, of tweets and users. To be precise, 5826,655 tweets shared by 783,185 unique users have been collected. The harvested tweets consist of 593,794 originals, 5116,265 retweets, 66,032 replies, and 50,564 quotes.

### Data repository

The proposed dataset is publicly available in Mendeley Data [5]. In the context of the 2019 Spanish general election (November 10th, 2019), the collection at hand reports a sample of Twitter’s traffic gathered from October 4th, 2019 to November 11th, 2019. All references to the tweets and the users are anonymized to guarantee the privacy of the accounts involved.

Data have been published in three formats to provide maximum flexibility, and they have been summarized in Table 1.

• JSON format. The dataset in plain JSON format was generated using the mongoexport utility. Both the users and the collections of tweets are available as JSONs.
• BSON format. The dataset in BSON format was generated using the mongodump utility. Besides the tweets’ and the users’ collections in binary BSON format, it also includes a file per collection with the related metadata required by the official tool to create direct indexes in the MongoDB.
• CSV format. The dataset in CSV comma separated format was generated using the mongoexport utility. It only includes the tweets’ collection due to the limitations of plain CSV, i.e., this data format does not include the users’ collections.

**Table 1:** Summary of data formats, sources, contents, and suggested restore utility.

Format	Tweets	Users	Import utility
JSON	✔	✔	mongoimport
BSON	✔	✔	mongorestore
CSV	✔	✘	mongoimport

### Usage

Users who want to use this dataset can freely access it. The easiest way is downloading the dataset by directly visiting the repository [5]. Please refer to the official MongoDB documentation for a full description [7]. To restore the data to a MongoDB instance, use the BSON data format as preferred source. However, both the JSON and the BSON data format would work fine.

**Fig. 1:**
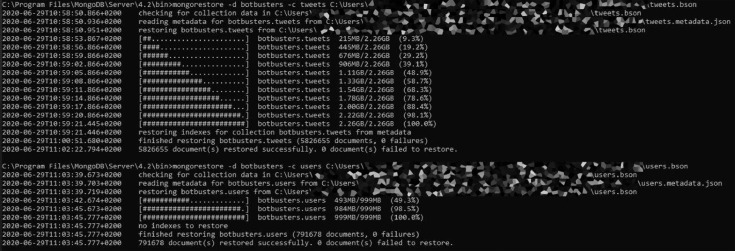
Import commands for the collections restoration using the MongoDB utility “mongorestore”.

To import using the BSON format, the standalone commands to use are as follows. Fig. 1 reports a sample run for the import phase.

• mongorestore -d botbusters -c tweets .\tweets.bson
• mongorestore -d botbusters -c users .\users.bson

**Fig. 2:**
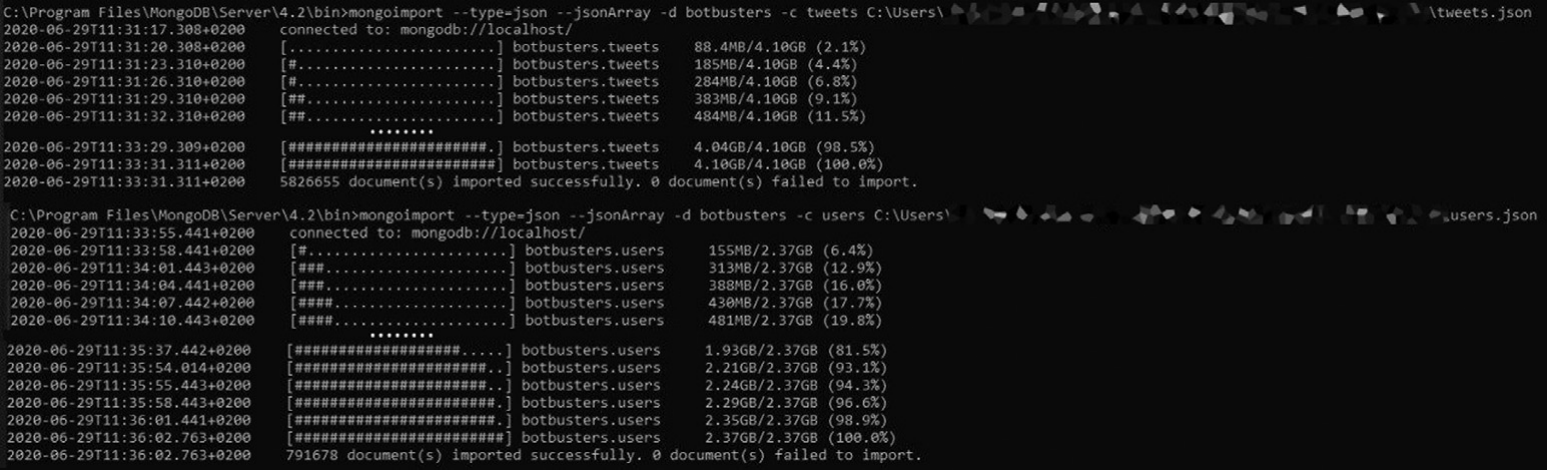
Import commands for the collections restoration using the MongoDB utility “mongoimport”. Notice the jsonArray flag.

To import using the JSON format, the standalone commands to use are as follows; notice the jsonArray flag. Fig. 2 reports a sample run for the import phase.

• mongoimport –jsonArray -d botbusters -c tweets .\tweets.json
• mongoimport –jsonArray -d botbusters -c users .\users.json

### Code repository

The code associated to this project is available on GitHub [6], documented to be easily followed and deployed. The different code files (Jupyter notebooks) stored in the repository are used to process, augment, and analyze the data. These are specifically listed in Table 2 together with a brief description. These notebooks are provided with a document (datacollection.md) that describes the supplementary materials that are necessary to understand the methodology and implementation of the experiments.

**Table 2:** Code files for the processing and analysis of data.

Filename	Main goal
Phase1.ipynb	Processing, refining, completing, and formating the raw collected data.
Phase2.ipynb	Data augmentation with calculated features and anonymization of personal properties.
Phase3.ipynb	Statistical analysis of the data regarding the social bots’ activities.
Phase4.ipynb	Feature engineering, analysis, and representation of users political classification.
Phase5.ipynb	Analysis and characterization of classified social bots activity and behavior.

### Figures, tables, formulas, and algorithms

In the rest of the paper, Table 3 compiles the list of hashtags used to harvest the tweets during the observation period; Table 4 describes the features contemplated for each identified user; Table 5 enumerates the features implemented for each extracted tweet; and Table 6 specifies the literal expressions used to build each defined tweet bag-of-words. Finally, Fig. 1 and Fig. 2 report the commands and expected results for the restoration process.

**Table 3:** List of hashtags used to harvest the tweets during the observation period.

Group	Keyword	Group	Keyword
VOX	#VOX	UP	#ElPoderDeLaGente
VOX	#EspañaSiempre	UP	#MamadasPodemos
PP	#PartidoPopular	UP	#SePuede
PP	#PP	UP	#UnGobiernoContigo
PP	#PorTodoLoQueNosUne	UP	#UnidasPodemos
Ciudadanos	#Cs	Elections	#10NElecciones
Ciudadanos	#Ciudadanos	Elections	#10Noviembre
Ciudadanos	#EspañaEnMarcha	Elections	#Elecciones10N
PSOE	#AhoraSí	Elections	#eleccionesgenerales10N
PSOE	#AhoraEspaña	Elections	#EleccionesNoviembre2019
PSOE	#PSOE	Elections	#10N
PSOE	#PSOEcompraVotos	Exhumation	#francisfrancoesp
Catalonia	#tsunamiinfiltrado	Exhumation	#FrancoCalientaQueSales
Catalonia	#116YA	Exhumation	#unboxingfranco
Catalonia	#disturbiosBarcelona	Exhumation	#exhumacionFranco
Catalonia	#EstadoDeExcepcion	Debate	#DebateA5
Catalonia	#MarlaskaDimisionYa	Debate	#Debatea7RTVE
Catalonia	#SpainIsAFascistState	Debate	#DebateElectoral
Catalonia	#ThisIsTheRealSpain	Debate	#DebatePresidencial
Catalonia	#tsunamidemocractic	Debate	#ElDebate4N
AbascalEH	#BoicotElHormiguero	Debate	#ElDebateEnRTVE
AbascalEH	#SantiagoAbascalEH	Debate	#UltimaOportunidadL6
AbascalEH	#elhormigueroabascal	Debate	#Debate10N

**Table 4:** Users’ features.

Feature	Description	Origin	Anon.	Domain
_id	It contains the user’s ID*^?^*.	Twitter	✔	UUID
scores.categories.content	Score indicating an analysis of the content and language of the user’s tweets^†^.	Botometer	✘	R ∈ [0,1]
scores.categories.friend	Score expressing how the user behaves with other users in terms of follower-friend relations and types of tweets^†^.	Botometer	✘	R ∈ [0,1]
scores.categories.network	Score representing the interconnections with other users^†^.	Botometer	✘	R ∈ [0,1]
scores.categories.sentiment	Score focusing on the emotion and attitude of the user’s tweets^†^.	Botometer	✘	R ∈ [0,1]
scores.categories.temporal	Score studying the behavior of the user along time^†^.	Botometer	✘	R ∈ [0,1]
scores.categories.user	Score analyzing the user’s metadata^†^.	Botometer	✘	R ∈ [0,1]
scores.scores.english	Overall classification results^†^.	Botometer	✘	R ∈ [0,1]
scores.scores.universal	Overall classification results without English-based features^†^.	Botometer	✘	R ∈ [0,1]
scores.cap.english	Probability that the user’s account is completely automated. It uses all six feature categories^†^.	Botometer	✘	R ∈ [0,1]
scores.cap. universal	Probability that the user’s account is completely automated. It excludes *sentiment* and *content* feature categories^†^.	Botometer	✘	R ∈ [0,1]
scores.display_scores.english	Value of *scores.scores.english* multiplied by 5^†^.	Botometer	✘	R ∈ [0,5]
scores.display_scores.universal	Value of *scores.scores.universal* multiplied by 5^†^.	Botometer	✘	R ∈ [0,5]
scores.display_scores.content	Value of *scores.categories.content* multiplied by 5^†^.	Botometer	✘	R ∈ [0,5]
scores.display_scores.friend	Value of *scores.categories.friend* multiplied by 5^†^.	Botometer	✘	R ∈ [0,5]
scores.display_scores.network	Value of *scores.categories.network* multiplied by 5^†^.	Botometer	✘	R ∈ [0,5]
scores.display_scores.sentiment	Value of scores.categories.sentiment multiplied by 5^†^.	Botometer	✘	R ∈ [0,5]
scores.display_scores.temporal	Value of scores.categories.temporal multiplied by 5^†^.	Botometer	✘	R ∈ [0,5]
scores.display_scores.user	Value of *scores.categories.user* multiplied by 5^†^.	Botometer	✘	R ∈ [0,5]
followers	List of Twitter users’ ID representing the followers of the user*^?^*.	Twitter	✔	UUID
friends	List of Twitter users’ ID representing the friends (i.e., followings) of the user*^?^*.	Twitter	✔	UUID

*^?^* Twitter [3], ^†^ Botometer [4], R: Real numbers.

**Table 5:** Tweets’ features.

Feature	Description	Origin	Anon.	Domain
_id	It contains the tweet’s ID?.	Twitter	✔	UUID
created_at	UTC time when the Tweet was created?.	Twitter	✘	Date
favorite_count	Approximately, how many times the Tweet has been liked by Twitter users?.	Twitter	✘	N
in_reply_to_status_id	If the tweet is a reply, it contains the original tweet's ID?.	Twitter	✔	UUID
in_reply_to_user_id	If tweet is a reply, it contains the original tweet's author ID?.	Twitter	✔	UUID
retweet_count	Number of times the tweet has been retweeted?.	Twitter	✘	N
retweet_or_quote_id	If the tweet is a retweet or a quote, it contains the original tweet's ID?.	Twitter	✔	UUID
retweet_or_quote_user_id	If the tweet is a retweet or a quote, it contains the original tweet's author ID?.	Twitter	✔	UUID
tweet_type	Type of tweet: original, retweet, reply, or quote?.	Twitter	✘	Text
user_id	The tweet’s author ID?.	Twitter	✔	UUID
sentiment_score	Sentiment of the tweet’s text. *t^sent^* ∈ [0*,*1] †.	Calculated	✘	R
keywords_summary.VOX	Boolean indicating if the text contains keywords related to VOX political party.	Calculated	✘	B
keywords_summary.PP	Boolean indicating if the text contains keywords related to PP political party.	Calculated	✘	B
keywords_summary.Ciudadanos	Boolean indicating if the text contains keywords related to Ciudadanos political party.	Calculated	✘	B
keywords_summary.PSOE	Boolean indicating if the text contains keywords related to PSOE political party.	Calculated	✘	B
keywords_summary.UP	Boolean indicating if the text contains keywords related to UP political party.	Calculated	✘	B
keywords_summary.Elections	Boolean indicating if the text contains keywords related to the general elections.	Calculated	✘	B
keywords_summary.Exhumation	Boolean indicating if the text contains keywords related to the exhumation of the fascist dictator Francisco Franco.	Calculated	✘	B
keywords_summary.Catalonia	Boolean indicating if the text contains keywords related to the riots in Catalonia.	Calculated	✘	B
keywords_summary.Debate	Boolean indicating if the text contains keywords related to the main electoral debate.	Calculated	✘	B
keywords_summary.AbascalEH	Boolean indicating if the text contains keywords related to the participation of Santiago Abascal (VOX) in the TV program ‘El Hormiguero’.	Calculated	✘	B

? Twitter [3]

† Sentiment algorithm [8], N: Natural numbers, R: Real numbers, B: Boolean.

**Table 6:** List of keywords used for the bag-of-words. Matches were case-insensitive and matched to the closes Unicode character (*e.g*. ‘á’ is equivalent to ‘a’).

Group	Keyword	Group	Keyword
VOX	VOX	PP	PartidoPopular
VOX	EspañaSiempre	PP	Partido Popular
VOX	Abascal	PP	PP
VOX	Santiago Abascal	PP	PorTodoLoQueNosUne
VOX	Santi Abascal	PP	Pablo Casado
Ciudadanos	Ciudadanos	PSOE	AhoraSí
Ciudadanos	Cs	PSOE	AhoraEspaña
Ciudadanos	EspañaEnMarcha	PSOE	PSOE
Ciudadanos	Albert Rivera	PSOE	PSOEcompraVotos
Ciudadanos	Rivera	PSOE	Pedro Sánchez
UP	UnidasPodemos	Elections	10N
UP	Unidas Podemos	Elections	10NElecciones
UP	ElPoderDeLaGente	Elections	10Noviembre
UP	MamadasPodemos	Elections	Elecciones10N
UP	SePuede	Elections	eleccionesgenerales10N
UP	UnGobiernoContigo	Elections	EleccionesNoviembre2019
UP	Pablo Iglesias	Exhumation	exhumacionFranco
Catalonia	116YA	Exhumation	francisfrancoesp
Catalonia	disturbiosBarcelona	Exhumation	FrancoCalientaQueSales
Catalonia	EstadoDeExcepcion	Exhumation	unboxingfranco
Catalonia	MarlaskaDimisionYa	Debate	Debate10N
Catalonia	SpainIsAFascistState	Debate	DebateA5
Catalonia	ThisIsTheRealSpain	Debate	Debatea7RTVE
Catalonia	tsunamidemocractic	Debate	DebateElectoral
Catalonia	tsunamiinfiltrado	Debate	DebatePresidencial
AbascalEH	SantiagoAbascalEH	Debate	ElDebate4N
AbascalEH	elhormigueroabascal	Debate	ElDebateEnRTVE
AbascalEH	BoicotElHormiguero	Debate	UltimaOportunidadL6

## Experimental design, materials and methods

### Scenario

To build this dataset, we collected tweets (original, retweet, reply, and quote) from 46 hashtags related to the 2019 Spanish general election, collected between October 4th, 2019 and November 11th, 2019, using the Social Feed Manager (SFM) [2]. We equally distributed these hashtags among the five main political parties taking part in the election (i.e., UP, PSOE, Cs, PP, VOX), considering for each one its acronym and slogans. Besides, we harvested hashtags common to all parties, such as those related to the elections in a general manner and the main electoral debate on Spanish TV, as well as specific events with high relevance for the elections, highlighting the riots in Catalonia and the exhumation of the Spanish fascist dictator Francisco Franco. It is important to note that we only considered tweets mentioning at least one of the previous hashtags. However, due to the limitations of the Twitter's standard APIs, we cannot guarantee the completeness of the data. The complete list of hashtags considered is indicated in Table 3.

Taking into consideration the unstructured nature of tweet data and the static structure of the data acquired from SFM, we stored the harvested data in a MongoDB instance. We first defined a collection of tweets T containing all the information returned by the Twitter APIs, where a single tweet is denoted as t∈T. A second collection, identified asU, includes a unique set of users extracted fromT, where a single user is represented asu∈U [1]. The complete set of objects stored for each collection is indicated in the following “Features extraction” section.

### Features extraction

This section illustrates the features extracted and included in the dataset, which can have a different origin. The first one is the Twitter’s standard search APIs and includes all relevant aspects acquired from the tweets and their users. The second one is Botometer [4], a tool used for the identification of social bots in Twitter that returns the likelihood that the account is a bot.

Finally, the features can come from different algorithms used to generate knowledge over the harvested data, such as the calculation of the sentiment analysis over the tweets’ text [8].

It is worth noting that, to guarantee the anonymity of the dataset, the users’ and tweets’ identifiers have been replaced with randomly generated UUIDs. Because of that, we indicate for each feature whether it has been anonymized, or not. Besides, the tweets’ text has been deleted after the extraction of all related features to ensure the anonymity of the dataset.

### Users’ features

Most of the features considered for the gathered users are extracted from Botometer. Despite we have stored in our dataset all features provided by the tool, the most relevant one for our work is the CAP Universal (scores.cap.universal), since it excludes specific aspects of the tweet’s language (in contrast to the CAP English feature, i.e., scores.cap.english). The complete list of features included for each user is indicated in Table 4.

### Tweets’ features

Focusing on the features extracted for each tweet, Table 5 shows the whole list considered in this work. It is important to highlight a differentiation between the features directly obtained from the Twitter's standard search API and those computed by us.

### Bag-of-words

To identify the tweet’s topic mention, we defined five different bag-of-words (sets of keywords) denoted as W^Γ^, equally distributed between the different events not specifically related to any particular party. That is to say, the 2019 Spanish general election, the exhumation of the fascist dictator Francisco Franco, the riots in Catalonia, the main electoral debate, and the participation of the political leader Santiago Abascal in the TV show ‘El Hormiguero’. We have also calculated if a tweet mentions any of the five main political parties participating in the election. To do that, we defined five bag-of-words, denoted as W^P^, where P ∈ ℙ ={UP, PSOE, Cs, PP, VOX}. The complete set of keywords is represented in Table 6.

### Limitations

There is a low number of articles and tools available to perform sentiment analysis in Spanish, and the algorithm used in the tweets’ collection is not performing as desired [8]. Since the analysis of Spanish sentiment is not mature nowadays, further research is needed to improve this classification procedure. Additionally, the data collection is made using the Social Feed Manager (SFM) [2] that intrinsically leverages the Twitter API, limiting the requests temporally to a 7-days’ time window and not guaranteeing the retrieval of all tweets that contain the targeted hashtags.

## Ethical requirements

The 2019 Spanish general election data distributed with this article is a non-commercial research. Despite Twitter’s terms for content redistribution stipulate special permissions to academic researchers sharing Tweet IDs and User IDs for non-commercial research purposes, the published data have been appropriately anonymized by either removing or randomly modifying every field that might be used to identify the users. This procedure is performed to prevent and avoid the inference of sensitive characteristics of individual users by third parties. Therefore, the authors ensure the protection of the users' financial status or condition, political affiliation or beliefs, racial or ethical origin, or religious or philosophical affiliation or beliefs.

Further information regarding Twitter’s data policies is available in the official documentation accessible at https://developer.twitter.com/en/use-cases/academic-researchers.

## Declaration of Competing Interest

The authors declare that they have no known competing financial interests or personal relationships which have, or could be perceived to have, influenced the work reported in this article.

## Appendix Supplementary materials

Image, application 1
